# Understanding contraceptive use in Pakistan: The role of individual and community contextual factors

**DOI:** 10.1371/journal.pone.0342157

**Published:** 2026-02-03

**Authors:** Asifa Kamal, Lamia Alyami, Sadaf Malik, Maryam Siddiqa, Muhammad Ijaz, Afza Rasul, Jamal Abdul Nasir

**Affiliations:** 1 Department of Statistics, Lahore College for Women University, Lahore, Pakistan; 2 Department of Mathematics, College of Sciences and Arts, Najran University, Najran, Kingdom of Saudi Arabia; 3 Department of Mathematics and Statistics, International Islamic University, Islamabad, Pakistan; 4 Department of Mathematics and Statistics, The University of Haripur-KPK, Haripur, Pakistan; 5 Department of Statistics, Government College University, Lahore, Pakistan; Research and Development Solutions, PAKISTAN

## Abstract

Key findings from the PDHS 2017−18 indicate that the total demand for family planning among married women in Pakistan was 52%. Research is needed to understand the persistently low contraceptive use in the country. This study aimed to identify the individual and community-level contextual factors that influence contraceptive use in Pakistan. Data were taken from all four waves of the Pakistan Demographic and Health Surveys (PDHS); 1990−91, 2006−07, 2012−13, and 2017−18. A multi-level logistic regression model was used to estimate the strength of the association between contraceptive use for both individual and community-level factors. The final adjusted model revealed that the likelihood of contraceptive use among women increased from 1991 to 2013 but slightly declined in 2017−18. Specifically, in 2006−07 PDHS, the adjusted odds ratio (AOR = 3.14) of contraceptive use was lower than that in 2012−13 PDHS (AOR = 4.33). In the 2017−18 PDHS, the AOR = 4.02 was slightly lower than that in 2012−13. Contraceptive use was found to be lower among uneducated, older women and those with a low wealth index. This study found that decisions about contraceptive use are shaped by not only individual-level factors but also by community-level factors. Increases in contraceptive use were associated with individual-level characteristics such as women’s age at first cohabitation, education, work status, parity, husband’s education, and wealth index. A major community-level factor contributing to increased contraceptive uptake was community-level education.

## Introduction

Pakistan’s health system had not fully met the expected Millennium Development Goals (MDGs) related to maternal mortality ratio and under-five/infant mortality rates in 2015 and, despite alignment with the Sustainable Development Goals (SDGs), progress toward the 2025 targets has remained limited based on recent trends [1]. Family planning is associated with one of the key indicators of sexual and reproductive health (SDGs 3.7 and 5.6) [2]. Child and maternal mortalities often occur due to untimely, unintended pregnancies and hazardous abortions. Unintended pregnancies are projected to be almost 3.8 million each year in Pakistan [3]. Maternal mortality can be reduced significantly if the unmet need for family planning is fulfilled. According to the latest Pakistan Demographic and Health Survey (PDHS), every 5th currently married woman in Pakistan encountered an unmet need for family planning [4]. Mortalities due to shorter birth intervals and high parity can be cut down by the use of contraceptive methods [4].

Attainment of demographic dividends through advancing economic growth, reduction in poverty, and enhancement in the well-being of people is possible with a potential decline in fertility, particularly through sustained investment in family planning that reduces unintended pregnancies and yields economic and health benefits [5,6]. In Pakistan, about 21% of married women said they were doubtful about using contraceptives in the future, while about 46% said they had no desire to utilize family planning methods [4]. Furthermore, 16% of men said they thought using contraceptives was immoral [4]. There is also a perception that the use of contraceptives is immoral and 16% of men reported the same assertion [4,7–10]. Pakistan’s rapid population growth suggests that there is a significant unmet need for family planning services [11]. If Pakistan reaches a goal of a 55% contraceptive prevalence rate, the overall fertility rate could drop to about three births per woman [12]. Despite of emphasis on family planning programs, the desired contraceptive prevalence rate is still not achieved in Pakistan compared with international counterparts. In comparison to Turkey (73%), Iran (73%), Bangladesh (62%), Morocco (63%), Egypt (60%), and India (54%), Pakistan has the lowest prevalence of contraception (34%) [13]. Pakistan is one of the nations in South Asia that started a family planning program [14]. Just 34% of married women reported taking any kind of contraception during the program’s 50 years of existence [4]. In Pakistan, the rate of contraceptive use has stagnated or declined, highlighting the need to explore the underlying reasons for the lack of progress on this key indicator [15]. Pakistan’s CPR is predicted to drop to 45% by 2025, which is lower than Bangladesh’s (67%) and India’s (54%) [16].

In the current study, national-level data from all waves of the Pakistan Demographic and Health Survey was used to study the trends in contraceptive use over time, which is scarce in the literature. Community norms regarding fertility preferences and family planning effect individual’s behaviors towards the use of contraceptives [17–20]. Communities also facilitate women in handling social barriers, cultural myths, and misconceptions that prevent them from adopting family planning methods [21–25]. In low- and middle-income countries, it was reported that contraceptive use was low among women who belonged to 35 years and older age group [26]. A study in Ethiopia concluded that the use of contraceptives was higher in Comparing women with primary or no education to those with secondary or higher education [27]. The odds of contraceptive use were high in Yemen among women who belonged to poorer, middle, richer or richest households as compared to those who belonged to the poorest households [28]. Ideal family size is another potential predictor that is related to the use of contraceptives in low and middle-income countries [5].

Several studies have used multilevel regression models to investigate the community and individual level determinants of contraceptive use. A study in Kenya concluded that a multilevel model gives valid estimates of the parameters instead of simple binary logistic regression [29]. Similarly, a study in Ethiopia recommended to use multilevel model over traditional regression models as it investigates the effect of all predictors at different levels (individual and community) on the outcome variable (contraceptive use) [30]. The present research puts an effort to explore the socio-economic (women’s education, husband’s education, women’s employment status, wealth index)), demographic (age, age at first cohabitation, no. of living children, child mortality experience, region, place of residence), and cultural/attitudinal factors (fertility intention measured through ideal number of children) behind the low uptake of contraception among women in Pakistan.

The current study focuses on the following points

1. To determine the individual level factors (Demographic and socioeconomic) for contraceptive use.
2. To determine the community level factors (Demographic and socioeconomic) for contraceptive use.

### Conceptual framework

**Fig 1:**
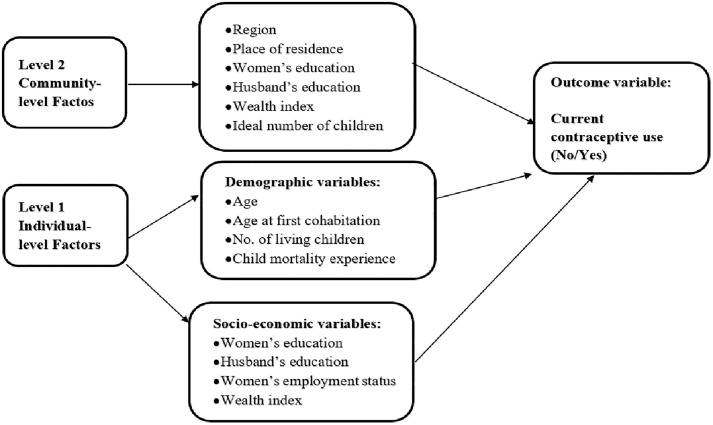
Conceptual framework illustrating individual- and community-level factors associated with contraceptive use.

Contraceptive use is affected by many individual and community-level factors. These factors were extracted from similar studies conducted in different regions and mentioned in the framework (Fig 1) [31–34]. The choices related to reproductive decisions depend greatly on the socio-cultural condition in which the individuals live [35]. Family and community contributions play an important role in decision-making than the individual’s decision [36–38]. Studies on community or contextual determinants of contraceptive use were considered important in research [39,40]. The community-level factors were used along with individual factors in the current study following the socio-ecological theory framework [41]. Fig 1 defines the conceptual framework of the study.

## Methods

### Data source and study design

Secondary data from the nationally representative Pakistan Demographic & Health Survey was used in this study and there was no need to take ethical approval. The data were accessed after permission from the demographic health survey program (https://dhsprogram.com/data/available-datasets.com) and access granted for the four waves of PDHS dataset. The data is in the open domain and accessible for everyone to replicate the analysis after permission from demographic health survey program. The complete terms of use are available on: https://dhsprogram.com/Data/terms-of-use.cfm.

Pakistan has only carried out four waves of these surveys from 1990–18, [4,42–44]. Data from all four waves were taken due to consistency in data collection methods and due to their relevance to the study objectives. In 1990−91, the contraceptive prevalence rate was only 12%. This percentage increased to 30% in 2006−07, reached 35% in 2012−13 and then slightly declined to 34% in 2017−18 [4].

A two-stage stratified clustered sampling design was utilized to choose the sample for each survey used in the current investigation. The sample was selected with the help of a sampling frame generated by the Pakistan Bureau of Statistics. In the PDHS sample, Primary Sampling Units (PSUs) were villages/mouzas for rural areas and enumeration blocks for urban areas. In the first stage, a total of 580 cluster (sample points) were selected that consisted of enumeration blocks by the method of probability proportional to their size. These enumeration blocks were the number of households residing in the specific block at the time of the survey. In the second stage, equal probability systematic sampling was used to select the fixed number of 28 households per cluster (enumeration blocks). The survey was successfully completed in 561 clusters after 19 clusters were dropped due to security concerns during the fieldwork. Sample sizes of four waves of PDHSs comprised 6611, 10023, 13558, and 15068 ever-married women of reproductive age 15−49, respectively (Table 1). The analysis was restricted to currently married and non-pregnant women. Moreover, Gilgit Baltistan and Islamabad (ICT) were not present in PDHS 1990−91 and PDHS 2006−07, therefore, these were excluded, and analysis was restricted to four provinces, i.e., Punjab, Sindh, Khyber Pakhtun Khwa (KPK), and Balochistan. As a result, only the four provinces are covered by the pooled data; wave-specific results, such as those from Islamabad, AJK, and Gilgit-Baltistan, are interpreted carefully for national generalizability. After imposing these two restrictions, sample sizes were reduced (Table 1). Table 1 defines unweighted counts unique to each survey wave, while multilevel analyses used individual-level survey weights to take stratification, clustering, and uneven probabilities of selection into account.

**Table 1:** Study Sample Size in the Four Waves of Pakistan Demographic and Health Survey (PDHS).

Survey	Total: 580 clusters Interviewed: 561 clusters 28 households per cluster			
	Number of women selected for interview	Number of women successfully interviewed	Response Rate (%)	Study Sample currently married and non-pregnant women
PDHS 1990−91	6904	6611	95.7	5357
PDHS 2006−07	10601	10023	95.0	8364
PDHS 2012−13	14569	13558	93.1	11343
PDHS 2017−18	15930	15068	94.6	10175

This study setting is significant because it can quantify the impact of independent factors (individual & community) on contraceptive use at the cluster level, where clusters may differ in social norms and environmental impacts due to the hierarchical nature of the data. Using Stata 14 melogit with robust standard errors, individual-level sample weights, stratification, and clustering at the PSU level were applied to all multilevel logistic regression models.

#### Outcome variable.

The response variable used in this study was “contraceptive use (modern or traditional) verses none” among the non-pregnant and currently married women of reproductive age (15–49). A dichotomous (binary) variable was created by categorizing women who reported using any contraceptive method (modern or traditional) as ‘Yes’ and those not using any method as ‘No’.

#### Independent variables.

The two primary categories of independent variables were individual-level factors and community-level factors. Individual level variables were taken directly from PDHS data, and they constitute the woman’s and her husband’s characteristics. These include age at the time of the interview, age at first cohabitation, number of living children, child mortality experience, education level, employment status, wealth index, women’s empowerment, and exposure to mass media.

The use of contraceptives is nested within the social and cultural context. Fertility intentions (community-level ideal number of children) and social development indicators (community level of wealth index and community level of education) at the community level have been computed and used in the analysis. Studies conducted at the country level that encompassed community level factors along with individual level factors furnished additional information about contraceptive practices [45]. Fertility intention measured from ideal family size and wealth index at the community level were found significantly associated with unmet need of contraception [45]. Another research that studied six countries reported that the adoption of contraceptives was affected by concepts and norms related to the reaction at the community level [46]. It was observed that regardless of personal viewpoints, the effect of the opinions of people living in the surroundings had a direct link with the use of contraceptives [47,48]. A Positive relationship between the education of spouses and the use of contraceptives has been commonly observed in many studies [45]. Education brings exposure and knowledge that changes behaviors, break traditional norms and societal pressures linked with the consumption of contraceptives [45]. Women living in communities with low levels of education affects the uptake of contraceptives due to less acceptability in the surroundings.

In this study, the individual-level characteristics for each PSU or cluster were combined to calculate community-level variables. By comparing each variable’s community-level value (cluster mean) with its national mean value, community-level variables were divided into high-level and low-level categories [21]. If the value was below the national mean value, it was categorized as low level and coded as 0 and if it was above the national mean value, then considered as high-level category and coded as 1. Region and place of residence were also added as community-level variables in the model. Women’s education, husband’s education, wealth index, and ideal no. of children were computed at the community level for the current analysis. These community level factor give information about community where women reside. For example, high level of wealth index at community level indicated that women belonged to community in which majority had on the average high wealth index. Data were analyzed using a complex survey setting in STATA 14 [49].

### Statistical model

After adjusting for women’s individual and household-level variables, a multi-level analytic strategy was driven to investigate the impact of community-level determinants on contraceptive use among young women using a nested data structure (i.e., women nested in communities). Community-level variables were created as cluster means of the raw individual-level measurements, and all individual-level continuous confounders (such as women’s age, wealth, and education) were group-mean centered at the cluster level. By distinguishing between-cluster (contextual) and within-cluster (individual-level) impacts, this method lessens the possibility of ecological and atomistic bias in cross-level interpretations.

Multi-level logistic regression was used due to the binary nature of the response variable. Traditional binary logistic regression model gives either underestimated or overestimated regression coefficients in the hierarchical nature of data as compared to multi-level models. It is because in traditional models standard errors of regression coefficients for cluster-level predictors are ignored and thus it results the serious bias in the β estimates of the parameters [50]. Multilevel models also help to estimate the cluster-level variables confounded with cluster-level dummy variables while in the traditional method it is not possible to isolate these effects due to observed and unobserved cluster characteristics [50].

The primary objective of multilevel logistic regression is to estimate the odds of occurrence of the event of interest while accounting for the hierarchical structure of the data. Accordingly, the multilevel mixed-effects logistic regression model is specified as follows:


$$\text{Log}\;\left[\frac{\pi \text{ij}}{\text{1}-\pi \text{ij}}\right]=\;\beta_{\text{0}}+\;\beta_{\text{1}}\;{\text{X}}_{\text{1ij}}+,\;\ldots ,+\;\beta_{\text{p}}\;{\text{X}}_{\text{nij}}+\;\mu_{\text{0j}}$$

where,

Here, $\pi_{\text{ij}}$ represents the probability that woman i in cluster j  is using contraception, while $\text{1}-\pi_{\text{ij}}$ denotes non-use. The term $\beta_{\text{0}}$ is the fixed intercept, $\beta_{\text{1}},\ldots ,\beta_{\text{p}}$ are coefficients for individual- and community-level predictors ${\text{X}}_{\text{ij}}$, and $\mu_{\text{0j}}$ is the random intercept capturing unobserved cluster-level variation. Given the binary nature of the outcome, a logit link function was used to model the relationship between predictors and the odds of contraceptive use [51].

Odds Ratio (OR) measures the fixed-effect part of the model, it computes the change in odds of the dependent variable due to one-unit change in the independent variable, holding effect of all other variables constant. A positive correlation with the result is shown by an odds ratio (OR) larger than 1, whereas a negative correlation is indicated by an OR less than 1. The intraclass correlation coefficient (ICC), proportional change in variance, and random intercept variance were used to evaluate cluster-level variation. The ICC computes the proportion of total variance in the dependent variable that is attributable to differences between clusters. Odds ratios computed when single independent and outcome variable was computed is called as unadjusted odds ratios (UOR) and when adjusted for all independent variables is called as adjusted odds ratio (AOR).

## Results

**Fig 2:**
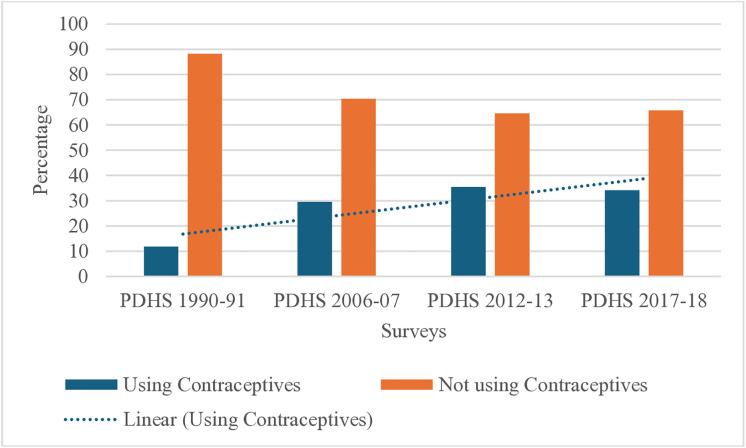
Pattern in Use of Contraceptive (modern or traditional) vs none among four waves.

It is evident from Fig 2 that majority Pakistani women did not utilize any kind of contraception, either traditional or contemporary. A descriptive increase in the percentage of contraceptive users is observed from 1991 to 2012–13; note that survey years are unevenly spaced, so the trend reflects observed survey data rather than assuming uniform yearly changes.

**Fig 3:**
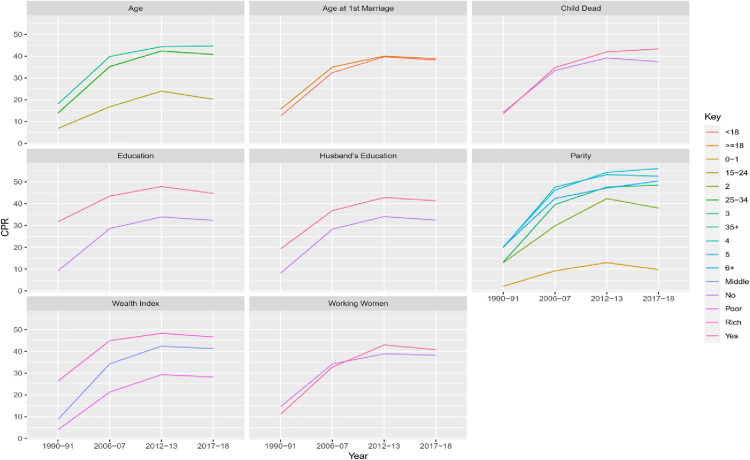
Patterns in Contraceptive Use (modern or traditional verses none) in Pakistan by Individual-level Characteristics.

The contraceptive prevalence was found to be higher among older, educated, high parity, and rich women. Women who had educated husbands also had higher contraceptive prevalence (Fig 3). The same trend persisted for different categories of individual-level factors across the four surveys. Contraceptive use increased with time till PDHS 2012−13 but showed a decline in PDHS 2017−18.

Little difference was found in the contraceptive use in PDHS 2012−13 and PDHS 2017−18 for child mortality. A nominal increase in contraceptive prevalence was observed for working women in the latter two surveys.

**Fig 4:**
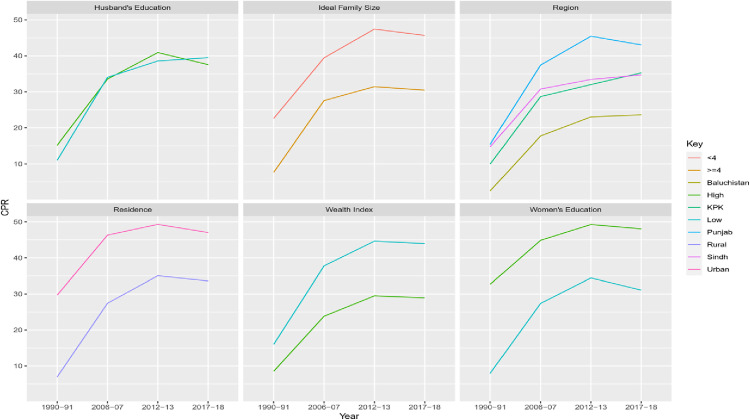
Patterns in Contraceptive Use (modern or traditional vs none) in Pakistan by Community-level Characteristics.

As far as the trend regarding contraceptive prevalence across different surveys is concerned, almost the same pattern was found for community-level factors as observed for individual factors (Fig 4). Decline was noted in the latest survey (PDHS 2017−18) for all community-level factors.

The contraceptive prevalence remained high for Punjab, Sindh, urban residents, communities with high women’s education, communities with low wealth index, and communities with low fertility intention except PDHS 2017−18.

All individual and community-level factors were significantly associated with contraceptive use in the unadjusted model (Table 2). These factors retained their significance when controlled for individual-level characteristics apart from child mortality (Model 1). All community-level factors had shown significant association with the current use of contraceptives in Model II. Husband’s education at the community level and child mortality lost their significance in Model III due to unmeasured community/cluster effects when controlled for all individual and community level factors. The magnitude of the effect slightly changed when factors were controlled at either the individual level (Model II) or both the individual and community level (Model III) but the direction/nature of the effect persisted the same. It was noticed that the period (survey year) was significantly related to the probability of contraceptive use. Women in the recent surveys (2007, 2013, and 2018) had greater chances of using contraceptive methods respectively as compared to women of reference period, i.e., 1991 (AOR = 3.14, CI: 2.84–3.48 for 2007; AOR = 4.33, CI: 3.93–4.76 for 2013; AOR = 4.02, CI: 3.64–4.43 for 2018). This is in line with other research showing that Pakistan’s use of contraceptives increased over time until 2012–2013. In comparison to younger women (15–24 years old), the chance of using contraceptives was significantly lower for women aged 25–34 and above 35 (AOR = 0.85 and AOR = 0.64, respectively), according to Model III’s results, which included both individual and community level characteristics. Compared to their counterparts (uneducated women), educated women had 1.66 times higher likelihood of using contraceptives. As the wealth index rose, so did the likelihood of using contraceptives. The likelihood of using contraceptive methods was 1.42 times higher for women in the medium wealth index category than for those in the poor category, and it rose to 1.80 times higher for women in the highest wealth index category.

**Table 2:** Results of Multilevel Logistic Regression Model Determining Factors Affecting the Use of Contraceptive Methods, (PDHS 1990−91 to 2017−18).

Characteristics	Null Model	Unadjusted Odds Ratio		Adjusted Odds Ratio					
				Model I		Model II		Model III	
				Individual Level Variables		Community-Level Variables		Individual & Community-Level Variables	
	Empty model	UOR (p-value)	95% CI	AOR (p-value)	95% CI	AOR (p-value)	95% CI	AOR (p-value)	95% CI
**Fixed effects**									
**Survey Year**	**(ref. = 1991)**								
2007	**---**	2.69	2.45-2.95*	3.13 (0.000)	2.84-3.45^*^	**---**	**---**	3.14 (0.000)	2.84-3.48^*^
2013	**---**	3.59	3.30-3.90^*^	4.38 (0.000)	3.99-4.80^*^	**---**	**---**	4.33 (0.000)	3.93-4.76^*^
2018	**---**	3.05	2.80-3.32^*^	3.81 (0.000)	3.47-4.18^*^	**---**	**---**	4.02 (0.000)	3.64-4.43^*^
**Age**	**(ref. = 15–24)**								
25-34	**---**	2.43 (0.000)	2.26-2.60^**^	0.91 (0.039)	0.83-0.99^*^	**---**	**---**	0.85 (0.002)	0.77-0.94^*^
35+	**---**	2.90 (0.000)	2.70-3.11^*^	0.73 (0.000)	0.67-0.81^*^	**---**	**---**	0.64 (0.000)	0.57-0.72^*^
**Women’s Education**	**(ref. = No)**								
Yes	**---**	2.07 (0.000)	1.98-2.17^*^	1.92 (0.000)	1.81-2.05^*^	**---**	**---**	1.66 (0.000)	1.55-1.78^*^
**Wealth Index**	**(ref. = poor)**								
Middle	**---**	1.63 (0.000)	1.53-1.75^*^	1.58 (0.000)	1.47-1.70^*^	**---**	**---**	1.42 (0.000)	1.30-1.54^*^
Rich	**---**	2.42 (0.000)	2.29-2.56^*^	2.32 (0.000)	2.16-2.48^*^	**---**	**---**	1.80 (0.000)	1.65-1.97^*^
**Parity**	**(ref. = 0–1)**								
2	**---**	4.59 (0.000)	4.20-5.02^*^	4.91 (0.000)	4.47-5.39^*^	**---**	**---**	4.98 (0.000)	4.48-5.54^*^
3	**---**	6.43 (0.000)	5.89-7.02^*^	7.97 (0.000)	7.22-8.79^*^	**---**	**---**	7.86 (0.000)	7.04-8.79^*^
4	**---**	7.98 (0.000)	7.30-8.72^*^	11.39 (0.000)	10.26-12.63^*^	**---**	**---**	11.32 (0.000)	10.08-12.72^*^
5	**---**	7.85 (0.000)	7.15-8.62^*^	13.08 (0.000)	11.70-14.64^*^	**---**	**---**	12.74 (0.000)	11.24-14.45^*^
6+	**---**	6.41 (0.000)	5.89-6.97^*^	13.50 (0.000)	12.09-15.07^*^	**---**	**---**	13.77 (0.000)	12.17-15.58^*^
**Age at 1**^**st**^ **cohabitation**	**(ref.= < 18)**								
>=18	**---**	1.06 (0.006)	1.01-1.11^*^	1.12 (0.000)	1.06-1.18^*^	**---**	**---**	1.14 (0.000)	1.07-1.21^*^
**Child mortality experience**	**(ref. = No)**								
Yes	**---**	1.06 (0.025)	1.02-1.11^**^	0.99 (0.860)	0.93-1.05	**---**	**---**	0.99 (0.808)	0.93-1.05
**Women’s Employment status**	**(ref. = No)**								
Yes	**---**	1.07 (0.014)	1.01-1.14^**^	1.13 (0.000)	1.06-1.21^*^	**---**	**---**	1.15 (0.000)	1.07-1.23^*^
**Husband’s Education**	**(ref. = No)**								
Yes	**---**	1.66 (0.000)	1.58-1.75^*^	1.16 (0.000)	1.09-1.23^*^	**---**	**---**	1.13 (0.000)	1.06-1.20^*^
**Community-level factors**									
**Region**	**(ref. = Punjab)**								
Sindh	**---**	0.77 (0.000)	0.71-0.82^*^	**---**	**---**	0.73 (0.000)	0.68-0.78^*^	0.76 (0.000)	0.71-0.83^*^
KPK	**---**	0.75 (0.000)	0.69-0.80^*^	**---**	**---**	0.84 (0.000)	0.78-0.90^*^	0.84 (0.000)	0.78-0.91^*^
Balochistan	**---**	0.41 (0.000)	0.37-0.46^*^	**---**	**---**	0.57 (0.000)	0.51-0.63^*^	0.56 (0.000)	0.50-0.63^*^
**Place of Residence**	**(ref. = Urban)**								
Rural	**---**	0.50 (0.000)	0.47-0.52^*^	**---**	**---**	0.66 (0.000)	0.62-0.70^*^	0.75 (0.000)	0.70-0.81^*^
**Women’s Education**	**(ref. = low level)**								
High level	**---**	2.52 (0.000)	2.39-2.65^*^	**---**	**---**	1.67 0.000)	1.56-1.79^*^	1.20 (0.000)	1.11-1.30^*^
**Husband’s Education**	**(ref. = low level)**								
High level	**---**	0.95 (0.055)	0.91-1.01^***^	**---**	**---**	0.92 (0.002)	0.88-0.97^*^	1.01 (0.498)	0.96-1.07
**Wealth Index**	**(ref. = low)**								
High	**---**	0.59 (0.000)	0.56-0.62^*^	**---**	**---**	0.59 (0.000)	0.56-0.63^*^	0.81 (0.000)	0.76-0.87^*^
**Ideal no. of children**	**(ref.=<4)**								
>=4	**---**	0.45 (0.000)	0.42-0.47^*^	**---**	**---**	0.71 (0.000)	0.66-0.75^*^	0.71 (0.000)	0.66-0.77^*^

*Significant at 1%, i.e., (P-value < α) where α = 0.01, **Significant at 5%, ***Significant at 10%.

Higher parity was observed to enhance the odd ratios of contraceptive use [52]. Women who cohabitated for the first time at or after the age of 18 were 1.14 times more likely to use contraception than women who cohabitated for the first time before the age of 18. Compared to women who had not suffered child mortality, those who had were less likely to utilize contraceptives. Women who were employed were 1.15 times more likely than jobless women to use contraceptive methods. Compared to women with uneducated husbands, those with educated husbands were 1.13 times more likely to use contraception.

In Model III, when controlled for both individual and contextual factors, it was found that variations persisted in the use of contraceptives for women who lived in Sindh, KPK, and Balochistan as compared to Punjab. It was 24% (AOR = 0.76), 16% (AOR = 0.84), and 44% (AOR = 0.56) less likely to use contraceptive methods for women residing in Sindh, KPK, and Balochistan as compared to women residing in Punjab. The rural community residents had (AOR = 0.75) 25% lower odds of contraceptive use as compared to urban community residents. A woman with a higher level of women’s education had 1.20 times more chances to use contraceptive methods than a woman with a lower level of education. Husband’s education at the community level, when controlled for individual and community level factors, had lost its significance. Women with high fertility ambitions (ideal family size of more than four) were less likely to use contraceptives than those with low fertility aspirations (ideal number of children of fewer than four) (AOR = 0.71).

The value of the estimated random intercept variance (0.32) indicated that significant variability was found in the use of contraceptives across the clusters (Table 3). When both individual-level factors and community-level factors were used in the model (Model III), the random intercept variance was reduced to 0.08. The Intra-class Correlation Coefficient (ICC) in the null model showed that 8.8% of the total variation found in contraceptive use was due to selected community-level variables. Model III’s reduced ICC revealed that the combined influence of individual-level and community-level variables accounted for 2.6% of the intra-cluster variation in contraceptive use. A low level of correlation between members of a particular cluster is indicated by a model with a small ICC [50]. According to Model 1’s Proportional Change of the Variance (PCV), the individual-level factors accounted for 56.2% of the variability in the cluster of contraceptive users. PCV of Model III revealed that both individual and community-level factors accounted for 75.0% of the overall variability associated with the usage of contraceptives. Even though Model III’s ICC was low, it was thought to be a better fit for contemporary contraceptive use due to the data’s hierarchical structure, high PCV value, and values of MOR, AIC, and BIC.

**Table 3:** Model Fit Statistics.

	Null Model	Model I	Model II	Model III
		Individual Variables	Community-Level Variables	Individual & Community-Level Variables
Random intercept variance	0.32	0.14	0.10	0.08
(ICC^a^ %)	8.8	4.2	2.9	2.6
(PCV ^b^%)	Reference	56.2	68.7	75.0
MOR^c^		1.3505	1.3703	1.3301
AIC^d^		40634.08	37944.18	33116.42
BIC^e^		40796.39	38027.93	33342.45

^a^Intra Cluster Correlation ^b^Proportional Change of the Variance ^c^Median Odd Ratio ^d^Akaike Information Criterion ^e^Baysian Information Criterion.

## Discussion

The present article focused on finding the individual and community-level factors associated with contraceptive use among currently married and non-pregnant women of Pakistan. This utilized data from all four waves of PDHS and explored the trends in contraceptive use from 1990−91–2017−18.

Contraceptive use was strongly correlated with all community-level characteristics, indicating that Pakistani women’s behavior was shaped by their communities. Notably, compared to communities with low fertility goals, women who lived in communities with high fertility intentions (ideal number of children ≥4) used less contraceptives (Fig 4). This emphasizes how important community norms about family size are in influencing people’s use of contraceptives. Women in communities with high fertility goals are impacted by the social expectations of the families in their immediate vicinity and may refrain from using contraceptives out of concern for social rejection [53]. The decision to utilize birth control methods is hampered by ideas spread by communal networks regarding high family sizes. Women’s choices are influenced by what is deemed “normal” or “ideal” in their community [54]. Such social norms are difficult to change, and programs aimed at them need years of consistent work. Long-term policy consistency is crucial because beneficial effects on community habits take time to manifest.

The community wealth index had a complicated impact. The wealth index and contraceptive use were shown to be positively correlated at the person level (AOR = 1.42 for middle-class and 1.80 for rich), but the link seemed to be inverse at the community level. This may reflect attitudinal behaviors embedded in cultural norms rather than affordability, as contraceptives are widely available free of cost (44% of women availed facility of free access) [55]. Recent evidence also suggests that middle-class households in Pakistan tend to have fewer children compared to both poor and rich households (Naz et al., 2024), highlighting that dichotomizing wealth at the national mean may oversimplify community-level effects.

Geographical and regional disparities were observed. The odds of contraceptive use among women residing in Sindh (AOR = 0.76), Balochistan (AOR = 0.56), and KPK (AOR = 0.84) were lower compared to Punjab [56]. The unmet need for family planning was highest in urban Balochistan (24%) and rural Sindh (22%) [4]. Rural dwellers had significantly lower contraceptive use (AOR = 0.75, 19% vs 15% in urban areas) [4]. Lack of exposure to family planning methods through media was also higher for Baloch men (70%) [4]. Urban residents had better access to education, healthcare, and information on contraceptives, which contributes to higher prevalence.

Women’s education at the community level was positively associated with contraceptive use (AOR = 1.20 for high level) and is linked to empowerment, enabling women to build social capital and make autonomous decisions [57–62]. Similarly, husbands’ education contributed to contraceptive uptake (AOR = 1.13 for high level), though the influence of community norms often mediated individual decision-making [63,64]. A noticeable proportion of educated Pakistani women reported awareness of family planning methods (34%), as documented in the PDHS 2017–18 [4].

Following the discussion of community-level effects, individual-level factors remain relevant. Contraceptive use decreased with age (AOR = 0.85 for 25–34, 0.64 for 35+), was higher among educated, employed, and wealthier women, and increased with parity (AOR up to 13.77 for 6 + children). Child mortality experience reduced contraceptive use, consistent with child hoarding theory [65–68]. However, the inclusion of community-level factors in the models adjusted individual-level effects only slightly, suggesting that community context plays a more substantial role than individual characteristics alone.

Family planning is regarded as a pillar to promote safe motherhood. Use of different contraceptive methods reduces unintended pregnancies and associated maternal and child complications [69]. To maximize impact, programs should prioritize interventions in communities with high fertility intentions and focus on changing social norms over the long term. Free service delivery, counseling, and sustained community engagement can gradually alter norms and improve contraceptive uptake. At individual level, particularly target older women (>44 years), women not engaged in economic activity, less educated, and newlywed couples for counselling to improve the uptake of contraceptives.

## Conclusion

This study found that decision about contraceptive use is shaped by not only individual-level factors but also by community-level determinants. Increasing trend is evident from 1990 to 2013, in the use of contraceptives, but thereafter a decline is observed. An increase in the use of contraceptives was attributable to individual-level characteristics like women’s age at first cohabitation, women’s education, women’s work status, parity, husband’s education, and wealth index. A major community-level factor that improved the uptake of contraceptives, was education. Education, both at individual and community levels, opened paths for adopting contraceptives. Rural dwellers and high fertility intention communities prevented women from the use of contraceptives. Lower prevalence of contraceptive use was observed in Sindh, KPK, and Balochistan as compared to Punjab, due to economic and socio-cultural drivers. The findings of this study may assist policymakers in initiating family planning initiatives at the local level. Motivating females to get involved in skill-based education and participate in the labour force is one of the key challenges for policymakers. All communities, irrespective of high or low wealth quintile, need to be sensitized about the use of contraceptives, particularly those who have high fertility intentions. It is recommended that future research should use Multi-level modeling over traditional regression models when analyzing DHS data, to reduce bias and get better estimated regression coefficients. The key strength of this study is the use of data from the Demographic and Health Survey Data sets, which is considered accurate and representative data over the 90 countries. Both individual and community level factors were accounted, and appropriate methodology, i.e., Multilevel models, were used.

### Limitations

There are some limitations of this study. Women who were pregnant at the time of the survey were not included. Due to the large percentage of missing values, some factors pertaining to fertility preferences and husband-related characteristics, such as the husband’s desire for additional children, were excluded from the study. Information on media exposure and women’s empowerment was not collected in the earlier PDHS rounds (1990–91 and 2006–07); therefore, these factors could not be included in the analysis for those survey waves. Therefore, the pooled analysis did not examine the impact of these two factors on the trend of contraceptive use. To ensure comparability of contraceptive use trends over time, three regions—Gilgit-Baltistan, Azad Jammu and Kashmir, and Islamabad Capital Territory (ICT)—were excluded from the pooled analysis because they were not included in the PDHS 1990–91 and 2006–07 surveys.

Furthermore, because the PDHS data were cross-sectional, it was impossible to determine the causal linkages between the response variable and regressors (individual-level and community-level factors). The only thing that was estimated was the degree of correlation between predictors and predictand. Moreover, although the current technique offers a clear and consistent framework for analysis over successive survey waves, the dichotomization of community-level variables may obscure small changes between clusters.

## Supporting information

S1 File Approva letter for data. (PDF)

S2 File Normality assumption. (DOCX)
