# Supplementary material for: Understanding contraceptive use in Pakistan: The role of individual and community contextual factors
Source: PLoS One. 2026 Feb 3;21(2):e0342157. doi: 10.1371/journal.pone.0342157 (PMC12867252; doi:10.1371/journal.pone.0342157)
Supplement: S1 File — (PDF) [file pone.0342157.s001.pdf]

Jan 28, 2019

Sadaf Malik
Lahore College for Women University
Pakistan
Phone: 0323-6664686
Email: sadafmalik1324247@gmail.com
Request Date: 01/25/2019

Dear Sadaf Malik:

This is to confirm that you are approved to use the following Survey Datasets for your registered research paper titled: "Assessment of Factors Affecting the Use of Contraceptive Method in Pakistan":

Pakistan

To access the datasets, please login at: https://www.dhsprogram.com/data/dataset_admin/login_main.cfm. The user name is the registered email address, and the password is the one selected during registration.

The IRB-approved procedures for DHS public-use datasets do not in any way allow respondents, households, or sample communities to be identified. There are no names of individuals or household addresses in the data files. The geographic identifiers only go down to the regional level (where regions are typically very large geographical areas encompassing several states/provinces). Each enumeration area (Primary Sampling Unit) has a PSU number in the data file, but the PSU numbers do not have any labels to indicate their names or locations. In surveys that collect GIS coordinates in the field, the coordinates are only for the enumeration area (EA) as a whole, and not for individual households, and the measured coordinates are randomly displaced within a large geographic area so that specific enumeration areas cannot be identified.

The DHS Data may be used only for the purpose of statistical reporting and analysis, and only for your registered research. To use the data for another purpose, a new research project must be registered. All DHS data should be treated as confidential, and no effort should be made to identify any household or individual respondent interviewed in the survey. Please reference the complete terms of use at: https://dhsprogram.com/Data/terms-of-use.cfm.

The data must not be passed on to other researchers without the written consent of DHS. Users are required to submit an electronic copy (pdf) of any reports/publications resulting from using the DHS data files to: archive@dhsprogram.com.

Sincerely,

Bridgette Wellington

Bridgette Wellington
Data Archivist
The Demographic and Health Surveys (DHS) Program
