# Supplementary material for: Understanding contraceptive use in Pakistan: The role of individual and community contextual factors
Source: PLoS One. 2026 Feb 3;21(2):e0342157. doi: 10.1371/journal.pone.0342157 (PMC12867252; doi:10.1371/journal.pone.0342157)
Supplement: S2 File — (DOCX) [file pone.0342157.s002.docx]

**Assumptions**

**Normality of Random Effects**

The normality assumption in multilevel models applies to the distribution of the random effects, not the residuals. After fitting the multilevel model. Random effects were predicted that are also called Best Linear Unbiased Predictors – BLUPs. The predicted values are conditional modes, not actual realizations of the random effects. Diagnostics plots i.e. histogram, quantile normal plot (Q-Qplot) and kernal density plot. All depicted that distribution of random effects follows normal distribution.
